# Spatial Clustering of Tuberculosis-HIV Coinfection in Ethiopia at Districts Level

**DOI:** 10.1155/2023/5191252

**Published:** 2023-01-13

**Authors:** Leta Lencha Gemechu, Legesse Kassa Debusho

**Affiliations:** Department of Statistics, College of Science, Engineering and Technology, University of South Africa, Johannesburg, South Africa

## Abstract

**Background:**

Tuberculosis (TB) is a preventable and treatable disease but it is the leading cause of death among people living with HIV (PLHIV). In addition, the emergence of the HIV pandemic has also had a major impact on TB incidence rates. There are studies in spatial patterns of TB and HIV separately in Ethiopia; there is, however, no information on spatial patterns of TB-HIV coinfection in the country at the districts level at least using yearly data. This paper, therefore, aimed at determining the spatial clustering of TB-HIV coinfection prevalence rates in the country at the districts level on an annual basis over a four-year period, 2015–2018.

**Methods:**

District-level aggregated data on the number of TB-HIV infections were obtained from the Ethiopian Federal Ministry of Health for 2015 to 2018. The univariate and bivariate global Moran's index, Getis-Ord *G*_*i*_^*∗*^ local statistic, a chi-square test, and a modified *t*-test statistic for Spearman's correlation coefficient were used to evaluate the spatial clustering and spatial heterogeneity of TB among PLHIV and HIV among TB patients prevalence rates.

**Results:**

The district-level prevalence rate of HIV among TB patients was positively and significantly spatially autocorrelated with global Moran's *I* values range between 0.021 and 0.134 (*p* value <0.001); however, the prevalence of TB among PLHIV was significant only for 2015 and 2017 (*p* value <0.001). Spearman's correlation also shows there was a strong positive association between the two prevalence rates over the study period. The local indicators of spatial analysis using the Getis–Ord statistic revealed that hot-spots for TB among PLHIV and HIV among TB patients have appeared in districts of various regions and the two city administrations in the country over the study period; however, the geographical distribution of hotspots varies over the study period. Similar trends were also observed for the cold-spots except for 2017 and 2018 where there were no cold-spots for TB among PLHIV.

**Conclusions:**

The study presents detailed knowledge about the spatial clustering of TB-HIV coinfection in Ethiopia at the districts level, and the results could provide information for planning coordinated district-specific interventions to jointly control both diseases in Ethiopia.

## 1. Background

Tuberculosis (TB) and human immunodeficiency virus (HIV) are two of the most severe public health problems worldwide. Prior to the global coronavirus (COVID-19) pandemic, TB was the single infectious disease that claimed more lives each year than HIV [1]. The two diseases are epidemiologically associated [2]. The two diseases appear to be directly associated both within the host [3] and at the population level [4], according to codynamics that have been observed.

Tuberculosis is a preventable and treatable disease; however, it is the leading cause of death among people living with HIV (PLHIV) claiming millions of lives each year [5]. The emergence of the HIV pandemic has also had a major impact on TB incidence rates [6]. The HIV pandemic has led to a high increase in the number of TB cases in developing countries, especially in Sub-Saharan Africa in the 1990s [7]. HIV affects TB epidemiology by altering the natural course of infection and increasing the risk of latent TB infection (LTBI) reactivation [8]. WHO estimates that those living with HIV are 20 times more likely than those who do not get active TB [9]. Ten million people had active TB worldwide in 2017, and 9% of them also had HIV [10]. A third of the 36.9 million HIV and AIDS patients worldwide who also have TB infection. The most severely affected area is Sub-Saharan Africa, which is home to 70% of all TB-HIV coinfected individuals in the world [11].

TB claimed the lives of 1.3 million individuals in 2017 and an extra 300,000 PLHIV died due to TB-related illness [9]. In 2017, 11% of people with TB-HIV coinfection died while undergoing treatment, which was about three times the level among other people with TB, 4% [10]. In 2018, an estimated 862,000 PLHIV worldwide fell ill with TB and TB accounting for some 251,000 people who died from HIV-associated TB, which is about a third of AIDS deaths [12]. In 2019, there were an estimated 815,000 incident cases of tuberculosis globally among PLHIV, and 55% of them were diagnosed and notified. In the same year, there were an estimated 210,000 TB-related deaths among PLHIV, about a 63% reduction compared to 2010, where TB-related death was 570,000 among PLHIV [5].

In Ethiopia, an estimated 16,000 PLHIV contracted TB in 2016. During this year, a routine National Tuberculosis Program (NTP) report indicates that 8,625 (about 54%) have been receiving cotreatment. Narrowing this gap will be made possible by programmes for HIV and TB that are better at locating HIV and TB cases. According to studies conducted in Ethiopia, TB incidence is higher in areas with high HIV prevalence [13, 14]. The national responses for the integrated TB and HIV collaboration efforts were amended in 2012 based on the WHO recommendation and with the goal of lowering both the burden of TB among PLHIV and the burden of HIV among TB patients. The National guidelines recommend functional TB-HIV collaborative mechanism at all levels, including at national, regional, subregional, or districts and health service facility levels.

However, in Ethiopia, the distribution and prevalence of TB vary across regions and across districts within a region [14–16]. Furthermore, research studies on the prevalence of TB-HIV coinfection have revealed that the coinfection varies widely in Ethiopia (e.g., [13, 17]) and has geographical clustering [18]. Other countries have reported making similar findings [19, 20]. Such variations of tuberculosis may have significant implications for regional and national health policies in that decision-makers, medical professionals, and public health experts may gain from the evidence that tailored context-specific interventions are required for those regions and communities that are most at risk. In addition, Ethiopia's HIV prevalence is not distributed evenly [21], with certain regions showing a higher prevalence than others. It is crucial to conduct a more thorough examination of the epidemiological patterns of TB-HIV coinfection at the district level in order to develop the most efficient measures that contribute to lowering the rates of TB and HIV transmission. A study shows that by giving priorities by allocating resources to high-risk areas and executing effective preventative measures, the pandemic can be successfully controlled [22].

Few studies have reported the epidemiology of TB-HIV coinfection at the Hospital level [23, 24] in Ethiopia. Alene et al. [18] have recently used three years of aggregated data to report the spatial distribution of TB-HIV coinfection at the national level. However, based at least on yearly data at the national level, we are not aware of any publication on the spatial distribution of TB-HIV coinfection.

Identifying districts where the burden of TB-HIV coinfection is concentrated and its spatial distribution at the district level may help to identify populations at higher risk of coinfection. For effective surveillance programs and resource allocation [25], including identifying areas where existing TB and HIV collaboration programs and integrated services need to be reinforced [18] and information on hot-spots and cold-spots (or high and low burden) areas is also necessary. This paper, therefore, aimed at determining the spatial clustering of TB-HIV coinfection prevalence rates in Ethiopia at the districts level on an annual basis over a four-year period, 2015–2018.

The remainder of the paper is structured as follows. The data are first described. The statistical techniques employed in this investigation are then described in detail. The Results section contains the outcomes of using these techniques on the study's data. Finally, the Discussion and Conclusion sections, respectively, provide a discussion on the study's findings and limitations, as well as conclusions and suggestions for additional research.

## 2. Materials and Methodology

### 2.1. Study Area

**Figure 1:**
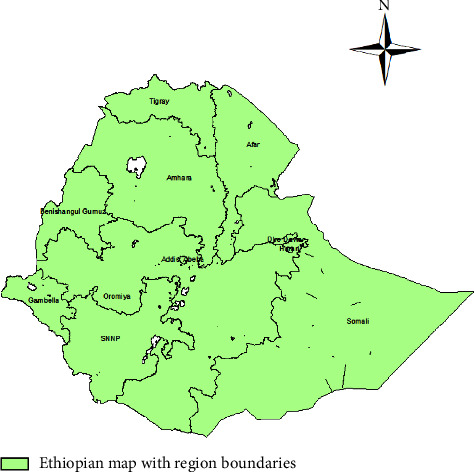
Map of Ethiopia with its nine regions and two city administrations.

Ethiopia is located in the North Eastern part of Africa. It shares borders with Eritrea to the North, Sudan in the west, Djibouti in the Northeast, Somalia in the east and Southeast, and Kenya in the south. The area of the country is roughly 1,127,127 square kilometres. Before 2020, the Federal Democratic Republic of Ethiopia has divided administratively into nine regional states (Tigray, Afar, Amhara, Oromia, Somalia, Benishangul-Gumuz, Southern Nations, Nationalities, and Peoples' (SNNP), Gambella, and Harari) and two city administrations (Addis Ababa and Dire Dawa). The map in Figure 1 displays these regions and city administrations. Each regional state is further divided into Zones, a zone into districts (called “woreda”), and a district into Kebeles (subdistricts). Public service delivery is now under the control of regional states due to the devolution of authority to regional administrations. The planning and delivery of services are the responsibility of the districts, while the administration of public health is the responsibility of the regional health bureaus. Districts are made up of a distinct population that is contained inside a specific administrative and geographic area. Districts have networks of primary health-care facilities, including district hospitals, health centers, and health posts. At the district level, health-related information is gathered and conveyed to the Zone Health Department, Region Health Bureau, and finally to the Federal Ministry of Health [16].

### 2.2. Data Sources

The Ethiopian Federal Ministry of Health provided district-level aggregate data from 2015 to 2018 on the number of TB patients enrolled in the national TB program, the number of TB cases tested for HIV, the number of TB patients with HIV-positive test results, the number of HIV patients who were screened for TB during their visit, the number of HIV patients with active TB and geographic coordinates (shape-files) of the country. The Federal Ministry of Health receives quarterly reports of these illnesses from the district health office via the Health Management Information System (HMIS) [26, 27]. In this paper, the spatial clustering of TB-HIV coinfection in Ethiopia at the districts level was investigated using these data. The variables of interest in this study were the prevalence of HIV among TB patients and the prevalence of TB among people living with HIV. These were calculated from the above data.

### 2.3. Statistical Analyses

This study used spatial analysis to determine where HIV prevalence among TB patients clustered geographically. This was carried out by dividing the number of TB cases with HIV-positive test results in a district by the total number of TB cases who were tested for HIV in that same district to determine the prevalence. The ratio of the number of TB cases with HIV-positive test results to the total number of HIV patients in the same district enrolled in HIV care facilities who were screened for TB during their visit was used to compute the prevalence of TB among PLHIV [18]. These are TB-HIV coinfection prevalence and their clusterings were obtained separately to assess the spatial patterns of the two types of coinfections at the districts level for each year in the study period. The geographic borders of the districts were geo-referenced for the spatial analyses and connected to the district data described in the Data Source section. Choropleth maps were created using ArcGIS software version 10.3 for display [28]. The statistical techniques applied in this paper are discussed below.

#### 2.3.1. Spatial Heterogeneity

Suppose *θ*_(hiv)*i*_ and *θ*_(tb)*i*_ are the true but unknown prevalence of HIV among TB patients and TB among people living with HIV, respectively, in district *i*, *i*=1,…, *n*=804, in Ethiopia, there are 804 districts at the time of data collection. The null hypothesis need to be tested that all districts have the same level of prevalence, say, prevalence of HIV among TB patients, i.e., *H*_0_ : *θ*_(hiv)1_=…=*θ*_(hiv)*n*_ against an alternative hypothesis that there is at least one district with a different risk level, i.e., *H*_1_ : *θ*_(hiv)*k*_ ≠ *θ*_(hiv)*l*_ for some *k* ≠ *l*. This test can be performed using a chi-square test [29]. Let *O*_*i*_ be the number of HIV cases among TB patients reported in district *i* and *n*_*i*_ be the number of TB patients in the same district. The maximum likelihood estimate of *θ*_(hiv)*i*_ is given by *O*_*i*_/*n*_*i*_. Then, the chi-square test statistic has the following form:

(1) $$\begin{array}{c} C=\overset{n}{\underset{i=1}{\sum }}\frac{{\left(O_{i}-E_{i}\right)}^{2}}{E_{i}}\text{,} \end{array}$$

where *E*_*i*_=*n*_*i*_ × (∑*O*_*i*_/∑*E*_*i*_) is the expected number of HIV cases among TB patients in district *i* assuming that HIV cases among TB patients arise at random across the country so that any district's share of the total number of HIV cases observed is proportional to the size of its own population at risk, i.e., number of TB patients. This assumption implies a constant prevalence or risk across the country. The test statistic measures an overall deviation from the constant prevalence. If the observed numbers for some of the districts are much different from the expected, then the test statistic *C* would become large, suggesting a departure from the constant prevalence assumption, which implies the presence of spatial heterogeneity in the number of HIV among TB patients. To assess statistical significance, when the number of districts *n* is large, which is the case for this study, the test statistic *C* follows a *χ*^2^ distribution with *n* − 1 degrees of freedom under the null hypothesis of a constant prevalence across the country. Therefore, we reject the null hypothesis *H*_0_ if *C* > *χ*_*n*−1_^2^ or if *p* value is less than 5% significance level.

#### 2.3.2. Univariate Spatial Pattern Analysis

The global pattern analysis to study the linear relationship between TB-HIV coinfection district-paired data was investigated using Spearman's correlation coefficient. The presence of spatially autocorrelation affects the variance of the test statistic *t*_0_ and hence the distribution of *t*_0_ under the null hypothesis *H*_0_ : *ρ*=0; for example, positive spatially autocorrelation increases the variance of *t*_0_ due to information loss ([29], Section 1.2.1). Therefore, we have applied the modified *t*-test of Clifford & Richardson [30]. In the modified *t*-test, the actual sample size *n* is replaced by the effective sample size *n*′ and hence the modified test statistic is given by $t_{0}=r\sqrt{n^{\prime }-2/1-r^{2}}$, which under the null hypothesis *H*_0_ : *ρ*=0 has *t*-distribution with *n*′ − 2 degrees of freedom. Thus, to make decision on the null hypothesis *t*_0_ was compared to the *t* distribution with *n*′ − 2 degrees of freedom.

Furthermore, the global spatial autocorrelation of TB-HIV coinfection separately and both types of coinfection prevalence rates simultaneously were investigated using the univariate global Moran's *I* [31] and bivariate global Moran *I* tests, respectively. Let *x*_*i*_ and *x*_*j*_ denote the observed values at districts *i* and *j*, *i*, *j*=1,…, *n*, where *n* is the number of districts. Then, the global Moran's Index or Moran's *I* [31] statistic is defined as follows:

(2) $$\begin{array}{c} I=\frac{\sum_{i=1}^{n}\sum_{j=1}^{n}w_{ij}\left(x_{i}-\bar{x}\right)\left(x_{j}-\bar{x}\right)}{W_{0}\sum_{i=1}^{n}{\left(x_{i}-\bar{x}\right)}^{2}}\text{,} \end{array}$$

where *W*_0_=∑_*i*=1_^*n*^∑_*j*=1_^*n*^*w*_*ij*_ is the aggregate of all the spatial weight with *w*_*ij*_ the weight is chosen according to the locations of *x*_*i*_ and *x*_*j*_. If the two observations are neighbors, *w*_*ij*_=1; otherwise, *w*_*ij*_=0. The simplest and commonly applied neighborhood definition is given by the binary connectivity matrix, as defined above or equivalently for two districts *i* and *j* it is defined as follows [32]:

(3) $$\begin{array}{c} w_{ij}=\left\{\begin{array}{cc} 1\text{,} & \text{if}\;\text{districts}\;i\;\text{and}\;j\;\text{share}\;a\;\text{boundary},\\ 0\text{,} & \text{otherwise}. \end{array}\right. \end{array}$$

Following this definition, since *w*_*ij*_=*w*_*ji*_ and *w*_*ii*_=0, which implies no self-correlation of an element with itself, hence the resulting spatial proximity matrix is necessarily symmetric.

In expression (1), when neighboring areas have similar values, where low values are close to low values and high values close to high values (or values are spatially clustered), it indicates positive spatial autocorrelation. But when neighboring areas incline to have different values (or spatially dispersed), it indicates negative spatial autocorrelation [32, 33] and the global Moran's *I* becomes close to 0 when some pairs of neighbors have the same direction of deviation and others have deviations in the opposite direction ([29], pp. 175).

#### 2.3.3. Local Moran's I Statistic

The global Moran's *I* statistic in expression (1) summarizes the spatial correlation with a single value. The local Moran's *I*, statistic introduced by [34], allows to assess the local spatial clustering (and local spatial outliers) and identify spatially correlated hot-spots. It is calculated for each observation. Let *x*_*i*_ be the *i* th observation at district *i*. Then, the local Moran's *I* statistic [34] is calculated using the following formula:

(4) $$\begin{array}{c} I_{i}=\frac{\left(x_{i}-\bar{x}\right)\sum_{j=1}^{n}w_{ij}\left(x_{j}-\bar{x}\right)}{{\hat{\sigma }}^{2}\sum_{j=1}^{n}w_{ij}}\text{.} \end{array}$$

An inference for a local spatial independence assumption may be based on the moments of statistic *I*_*i*_ or using a conditional randomization approach, however as the simulation study of [34] shows the latter provides a reliable basis for the inference.

#### 2.3.4. Getis-Ord *G*_*i*_^*∗*^ Statistic

The Getis–Ord *G*_*i*_^*∗*^ local statistic is an alternative to the local Moran's *I* index to determine the type of spatial cluster, i.e., hot-spot or cold-spot [35]. It is computed according to the following formula:

(5) $$\begin{array}{c} G_{i}^{*}=\frac{\sum_{j=1}^{n}w_{ij}x_{j}-\bar{x}\sum_{j=1}^{n}w_{ij}}{S\sqrt{\left[n\sum_{j=1}^{n}w_{ij}^{2}-{\left(\sum_{j=1}^{n}w_{ij}\right)}^{2}\right]/\left(n=1\right)}}\text{,} \end{array}$$

where *x*_*j*_ is disease count at district *j*, *w*_*ij*_ is a spatial weight that defines neighboring administrative districts *j* to *i*, *n* is the total number of districts, $\bar{x}=\sum_{j=1}^{n}x_{j}/n$, and $S=\sqrt{\sum_{j=1}^{n}x_{j}^{2}/n-{\bar{x}}^{2}}$, i.e., standard deviation.

Assuming that *G*_*i*_^*∗*^ is approximately normally distributed [36], *G*_*i*_^*∗*^ can be calculated as a standard normal variant with an associated probability obtained from the standard normal distribution [37]. Depending on the choice of confidence level (e.g., 90%, 95%, or 99%) on a map, clusters with a 90/95/99 percent significance level from a two-tailed normal distribution indicate significant clustering, i.e., cold-spots and hot-spots. A cluster of high values is suggested by districts with a high positive *z*-score and a small *p* value for a particular confidence level, with a larger *z*-score signifying a more intense cluster, or hot-spot. A lower *z*-score indicates a more intense cluster, or cold-spot, while districts with a low negative *z*-score and a small *p* value indicate there is a cluster of low values. Unlike the local Moran's *I* statistic, the Getis–Ord *G*_*i*_^*∗*^ statistic computed for each district is readily expressed in terms of *z*-scores [38], thus allowing for a more direct interpretation for statistical significance. In addition, more than one confidence intervals can be presented for it on a map and they are very visual. Therefore, in this paper, to identify the hot-spots and cold-spots districts for the prevalence of TB-HIV coinfection, i.e., prevalence of HIV among TB patients and the prevalence of TB among PLHIV, we applied the Getis–Ord *G*_*i*_^*∗*^ statistic.

### 2.4. Bivariate Local Moran's *I*

The spatial methods discussed above, i.e., global Moran's *I*, local Moran's *I*, and Getis–Ord *G*_*i*_^*∗*^ local statistics, only quantify the spatial structure of one variable at a time. The bivariate local Moran's *I* statistic is defined by the following expression:

(6) $$\begin{array}{c} I_{Biv}=\frac{\sum_{i}\left(\sum_{j}w_{ij}x_{i}\times y_{j}\right)}{\sum_{i}x_{i}^{2}}\text{,} \end{array}$$

where *x*_*i*_ is the first variable at location *s*_*i*_, e.g., tuberculosis among PLHIV, *y*_*j*_ is the second variable at each neighboring location *s*_*j*_, e.g., HIV among tuberculosis patients and *w*_*ij*_ is a spatial weight that defines neighboring administrative districts *j* to *i*. The bivariate local Moran's *I* statistic describes the simultaneous occurrence and hence coclustering of two diseases in space. Furthermore, as discussed in [39] it also describes a statistical relationship between a variable at a location and a spatially lagged second variable at neighboring locations.

A spatial weight matrix was used in this study to establish the spatial relationships between the districts, and Queen's contiguity was used to identify neighborhoods. Neighboring districts are those that share boundaries or a common vertex. Whereas the weight matrices for the local indicators of spatial association (autocorrelation) or for local Moran's *I* statistics, were defined using the nb2listw function from spdep [40] R package. The Clifford & Richardson modified *t*-test was done using the function modified *t*-test in the SpatialPack [41] R package. The significance of Moran's *I* statistics are examined through the use of the Monte Carlo randomization approach.

### 2.5. Ethical Consideration

The School of Science Ethics Committee at the University of South Africa granted permission for the study (ERC Reference Number: 2021/CSET/SOS/045). In addition, the Ethiopian Ministry of Health Office gave its consent for the study's data to be used. Because we used aggregated district-level data in this investigation, informed consent from the study participants was not obtained.

## 3. Results

### 3.1. Descriptive Statistics

The total number of TB patients enrolled in the national TB program, the total number of TB cases tested for HIV, the total number of TB patients with HIV-positive test results, the total number of HIV patients who were screened for TB during their visit, and the total number of HIV patients with active TB in Ethiopia stratified by gender and year are presented in Table 1.

In 2015, a total of 91,382 TB patients who were tested for HIV were enrolled to the Directly Observed Therapy Short Course (DOTS) and 623,927 HIV patients who were screened for TB during their visit were enrolled in HIV care. These numbers in 2016 were 91,692 and 914,987, in 2017 were 93,317 and 1,041,330, and in 2018 they were 52,402 and 719,651. All these reported cases were considered in this study. Except for those districts where their TB or HIV data had not available, the data used to cover all regions, zones, and districts of Ethiopia. From a total number of 91,382 TB cases enrolled to DOTS and who were tested for HIV in Ethiopia in 2015, about 54.66% (*n*=49,949) were male. Similarly, in 2016, 2017, and 2018, a higher proportion of TB cases enrolled to DOTS and who were tested for HIV were male, about 54.31% (*n*=49,802), 55.26% (*n*=51,564), and 55.32% (*n*=28,991), respectively. However, in the years 2015, 2016, 2017, and 2018, consistently higher proportions of patients enrolled in HIV care who were screened for TB were female 61.67% (*n*=384,788), 61.95% (*n*=566,839), 62.58% (*n*=651,680), and 62.91% (*n*=452,703), respectively (Table 1).

**Figure 2:**
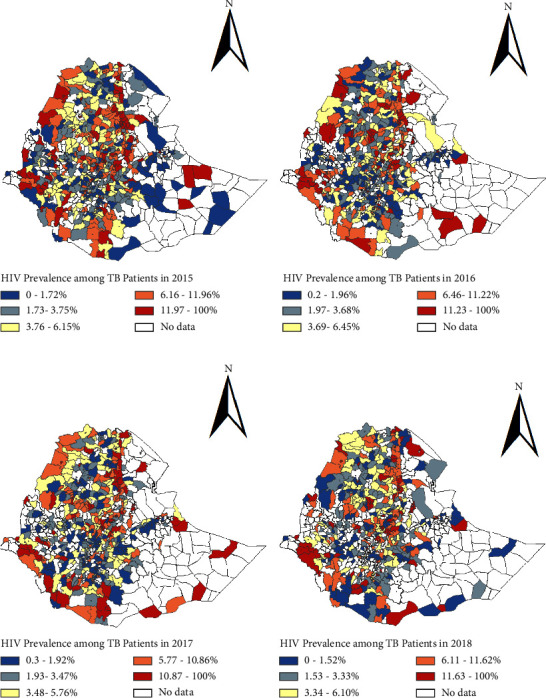
Geographical distribution of the prevalence of HIV among tuberculosis patients in Ethiopia in (a) 2015, (b) 2016, (c) 2017, and (d) 2018.

The prevalence of HIV among TB patients, denoted by HIV(TB), and the prevalence of TB among HIV patients, denoted by TB(HIV), in Ethiopia by year in all regions and two city administrations for the study period are given in Table 2. In 2015, the highest prevalence of HIV among TB patients was reported in the Gambella region (31.59%), whereas in the three years that follow 2016, 2017, and 2018 highest prevalence was reported in Addis Ababa city administration which were 24.98%, 22.40%, and 20.76%, respectively (Table 2). The results in Table 2 also show that the highest prevalence of TB among people living with HIV in 2015 was reported in the Somali region (100%); however, except for one district the reports from the other districts in this region were either reported as zero or not available. In 2016 and 2017, the prevalence for this region were 21.60% and 28.57%, respectively; and in both cases, the values were computed from two districts. In 2018, the highest prevalence was reported in the Gambella region (1.44%) (Table 2; Figure 2). The overall prevalence of HIV among TB patients in Ethiopia in 2015, 2016, 2017, and 2018 was 7.86%, 7.37%, 6.73%, and 6.88%, respectively, whereas the overall prevalence of TB among people living with HIV in 2015, 2016, 2017, and 2018 was 0.56, 0.42, 0.35, and 0.38 percent, respectively. According to the aforementioned findings, TB and HIV prevalence vary geographically over time. This will be further discussed in the sections that follow.

**Figure 3:**
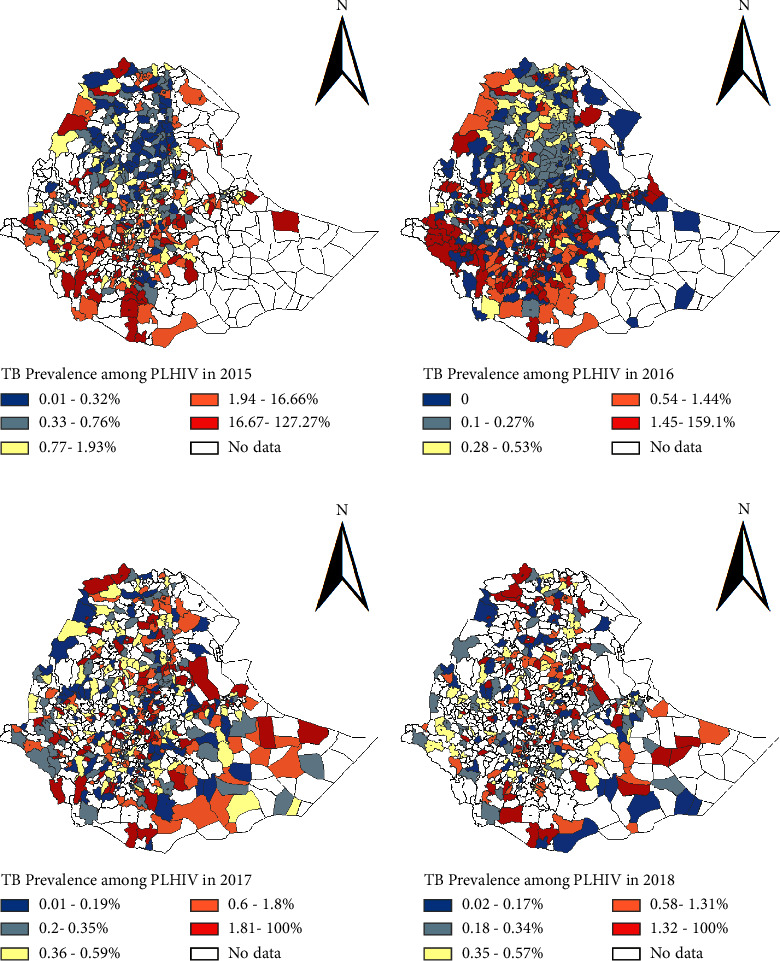
Geographical distribution of the prevalence of tuberculosis among people living with HIV in Ethiopia in (a) 2015, (b) 2016, (c) 2017, and (d) 2018.

However, Figures 2 and 3 show the observations that were made at a regional level are not necessarily reflected at the district levels. For example, in the year 2015, a high prevalence of HIV among TB patients at the district level was reported in various districts of Amhara, Benishangul-Gumuz, Gambella, Oromiya, and SNNP regions. Similarly, in 2016, high prevalence of HIV among TB patients was spotted in districts of Afar, Amhara, Benishangul-Gumuz, Gambella, Oromiya, SNNP, and Tigray regions. Furthermore, the high prevalence was also reported in Addis Ababa and Dire Dawa city administrations. Similar observations can be made from Figure 2 for 2017 and 2018.

In the case of TB among people living with HIV (Figure 3), the high prevalence was also reported in some districts of Addis Ababa city administrative, Afar, Amhara, Benishangul-Gumuz, Gambella, Oromiya, SNNP, Somali, and Tigray regions (Figure 3); like 2015, in 2016, high prevalence was reported in some districts of eight regions and the two city administrations. Except for Afar, Harar, and Gambella regions in 2017 and Harar, Gambella, and Dire Dawa city administration in 2018, there were some districts in other regions and Addis Ababa city administration which were identified as areas with a high prevalence of TB among PLHIV (Figure 3).

### 3.2. Spatial Heterogeneity in the Number of TB-HIV Coinfection

The chi-square tests on spatial heterogeneity in the number of HIV cases among TB patients had large test statistics of 9224.158, 8150.445, 7972.045, and 3964.088 for years 2015, 2016, 2017, and 2018, respectively. The same test for TB cases among people living with HIV had large test statistics of 67018.41, 30388.34, 22000.47, and 26112.08 for years 2015, 2016, 2017, and 2018, respectively. These large test statistics result in *p* values that are very close to 0, suggesting strong evidence against the null hypothesis of a constant prevalence of HIV among TB patients and TB among people living with HIV across Ethiopia, which agrees with the findings in the previous section. However, this test does not account for spatial data features, such as spatial autocorrelation of HIV cases among TB patients and TB cases among people living with HIV, this is discussed in the following section.

### 3.3. Spatial Analyses

#### 3.3.1. Spatial Clustering of TB and HIV Coinfections in Ethiopia

Table 3 presents the global Moran's *I* statistics of both data, i.e., HIV cases among TB patients and TB cases among people living with HIV, and Clifford & Richardson [30] modified *t*-test for spatial association of the two data sets for the study period. The modified *t*-test incorporates the spatial arrangement of the districts using the coordinates of the centroids.

The results in Table 3 show that for some of the study years there were evidences of spatial clustering of TB-HIV coinfection, in 2015 and 2017 with a Global Moran's *I* = 0.069 (*p* value <0.001) and 0.037 (*p* value <0.001) for the prevalence of TB among people living with HIV (PLHIV), respectively. Whereas for the HIV prevalence among TB patients Global Moran's *I* for 2015, 2016, 2017, and 2018 were 0.118, 0.134, 0.021, and 0.067, respectively, all with *p* value <0.001. The positive Global Moran's *I* values in Table 3 demonstrate that TB prevalence among PLHIV tends to be similar in any two spatially adjacent districts except in the year 2018 where a Global Moran's *I* value for the prevalence of TB among PLHIV was a small negative number close to zero (−0.001) with associated *p* value of 0.93 > 0.05, suggesting neighboring districts inclined to have spatially dispersed prevalence in the opposite directions. However, the dispersion was statistically nonsignificant.

The global Moran's *I* is consistently positive across the years for the prevalence of HIV among TB patients, indicating that over the study period of 2015–2018, any two spatially adjacent districts tend to have a similar prevalence of HIV among TB patients. Observe from Table 3 that except in 2017, the prevalence of HIV among TB patients was more spatially correlated than the prevalence of TB among PLHIV. Spearman's correlation coefficient values in Table 3 are positive and the Clifford & Richardson [30] modified *t*-test for spatial association show that TB among PLHIV and HIV among TB patients were significantly spatial associated (*p* < 0.001). The correlation varied with time, where the highest correlation was observed in year 2016 (*r*=0.692) and the lowest was in year 2018 (*r*=0.517). The global bivariate Moran's *I* was also consistently positive across the years suggesting that the district-level prevalence rates of TB among PLHIV were positively influenced by the prevalence of HIV among TB patients in the neighborhood districts and vice versa.

**Figure 4:**
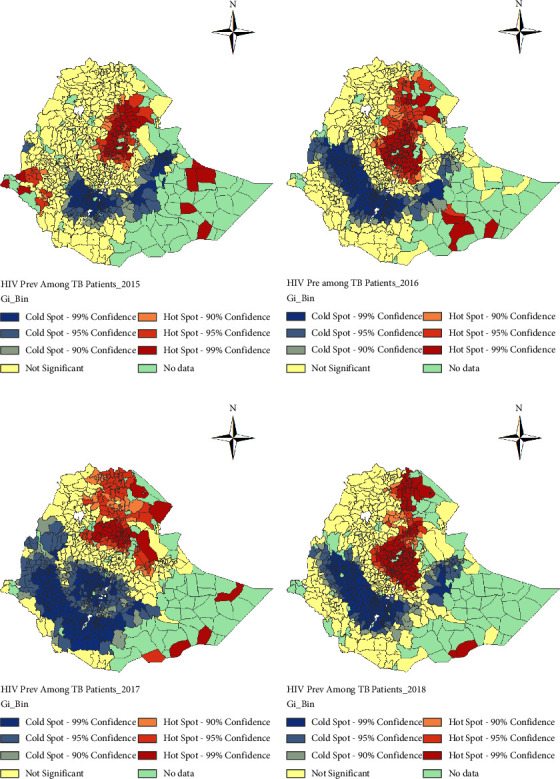
Spatial clustering of HIV prevalence among tuberculosis patients at district levels in Ethiopia from 2015 to 2018 using the Getis–Ord *G*_*i*_^*∗*^ statistic. (a) 2015. (b) 2016. (c) 2017. (d) 2018.

**Figure 5:**
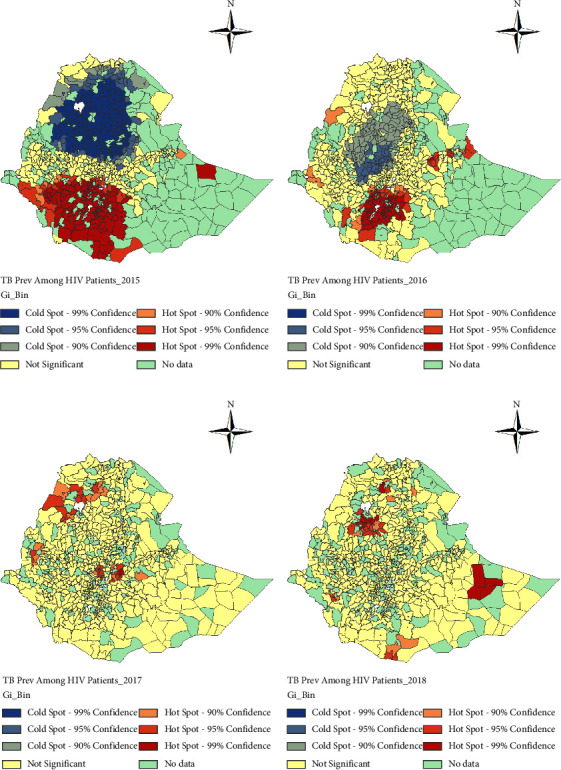
Spatial clustering of tuberculosis prevalence among PLHIV at district levels in Ethiopia from 2015 to 2018 using the Getis–Ord *G*_*i*_^*∗*^ statistic. (a) 2015. (b) 2016. (c) 2017. (d) 2018.

*(1) Spatial Clustering of TB-HIV Coinfections*. To further investigate the spatial heterogeneity, i.e., to identify the hot-spots and cold-spots districts for the prevalence of HIV among TB patients and for the prevalence of TB among PLHIV, we applied the Getis–Ord *G*_*i*_^*∗*^ statistic. Maps showing the distribution of spatial clusters of the prevalence of HIV among TB patients and the prevalence of TB among PLHIV are presented in Figures 4 and 5 for the study period, respectively.

The Getis–Ord *G*_*i*_^*∗*^ statistics in Figure 4 demonstrate that in 2015, districts in the Afar, Amhara (South and North Wolo zones), Gambela, Oromiya (West Oromiya), and Somali regions, as well as the Addis Ababa city administration, had hot-spots for the prevalence of HIV among TB patients in Ethiopia. The cold-clusters were concentrated around and in Dire Dawa city administration, Harer, parts of central and east Oromiya, and in SNNP regions. In 2016, hot-spots were also observed in districts of Afar, Amhara, Somali (south-west part), and Tigray regions, in Addis Ababa city administration, and districts of Oromiya around Addis Ababa. Whereas the cold-spots were concentrated in districts located in Benishangul-Gumuz, Oromiya, SNNP, and Harer regions. In 2017, districts in the Afar, Amhara, Somali, and Tigray regions had hot-spots for HIV among tuberculosis patients, whereas districts in the Benishangul-Gumuz, Gambela, Oromiya, and SNNP regions had cold-spots. Similar to 2017 in 2018, hot-spots were observed in districts located in Afar, Amhara, Oromiya, Somali, and Tigray regions and further in Addis Ababa city administration whereas the cold-spots for HIV among tuberculosis patients were concentrated in districts of Benishangul-Gumuz, Harer, Oromiya, and SNNP regions and in Dire Dawa city administration (Figure 4).

Figure 5 shows that in 2015 high clusters for the prevalence of TB among PLHIV or hot-spots were largely occurred in districts located in Gambela, Oromiya, and SNNP regions. In addition, a hot-spot was also observed in the Dagahbur zone around the Durkhsi border area in the Somali region. Whereas in the same year, the low clusters (cold-spots) for the prevalence of TB among PLHIV were observed in Afar, Amhara, and Tigray regions, and Addis Ababa city administration. On the other hand, in 2016 the hot-spots for the prevalence of TB among PLHIV were observed in Benishangul-Gumuz, Gambela, Oromiya, SNNP and Somali regions, and Dire Dawa city administration, whereas some of the districts in Amhara (north and east shewa zones) and Oromiya (north and west Oromia zones) regions had cold-spots. Relatively few hot-spots for prevalence of TB among PLHIV were observed in 2017 and 2018; however, there was no cold-spot observed in both years. In 2017, the hot-spots were observed in a few districts of Benishangul-Gumuz, Amhara (north Gondar and west Gojjam), Oromiya (districts around Addis Ababa and west Harerge zone) regions, and Addis Ababa city administration. On the other hand, in 2018 the hot-spots were observed in districts of Afar, Amhara, Oromiya (Borena zone, specifically Moyale district which is bordering with Kenya), and Somalia regions (Figure 5).

## 4. Discussion

Despite improvements in TB and HIV eradication due to WHO-coordinated efforts, these illnesses nevertheless have alarming mortality and morbidity statistics [1, 42]. For a four-year period (from 2015 to 2018), we looked at the spatial clustering of TB among HIV-positive individuals, i.e., PLHIV, and the prevalence of HIV among TB patients (i.e., TB-HIV coinfection) in Ethiopia at the districts level.

The prevalence of HIV among TB patients and the prevalence of TB among PLHIV varied between regions and between districts in Ethiopia over the study period. The chi-square tests on spatial heterogeneity in the number of HIV cases among TB patients have also supported these findings where the tests rejected the null hypothesis of a constant prevalence of HIV among TB patients and TB among PLHIV across Ethiopia.

The current study's results also demonstrate that over time, there were geographical differences in the prevalence of TB-HIV coinfection. These findings agree with those of Alene et al. [18], who found that the prevalence of TB among HIV-positive individuals and the prevalence of HIV among TB patients differed at the district level in Ethiopia. However, our data differ from theirs. The results from LISA using Getis–Ord *G*_*i*_^*∗*^ statistics show that the prevalence of TB among PLHIV was spatially clustered; i.e., there are hot-spots in districts of Gambela, Oromiya (specifically districts in Arsi, Borena, and Jimma zones), SNNP, and Somalli (in Liben and Deghabour zones) regions in 2015. However, unlike in 2015, in the other years, relatively few hot-spot districts were observed. This may be because of the wide implementation of the TB-HIV collaborative activities at the health facilities [43]. In 2015, the cold-spots for TB among PLHIV were observed in Afar, Amhara, Tigray regions, and Addis Ababa city administration. Similar to hot-spots, the number of cold-spots districts was reduced in 2016 and there was no cold-spot observed in 2017 and 2018. The result of 2018 could be due to a sharp decrease in the number of TB case notifications to the national HMIS from 2017 to 2018 (Table 1 and Figure 3(d)). About 12.3% of districts did not report in the number of TB case notifications to the national HMIS in 2018. This might affected the spatial patterns of the prevalence of TB among PLHIV in the year 2018 compared to the other years.

In this study, we have noticed that the hot-spots for the prevalence of TB among PLHIV have appeared in the country over the study period more in urbanized areas such as Awassa, Bahir Dar, Gondar, Jimma, and Shashemene. This may be connected to population movement within a district or between districts that are adjacent in search of employment or better living conditions. Studies conducted in Ethiopia have shown that spatial clustering of TB related to migration [44–49] and overcrowded and congested urban areas have high ongoing TB transmission [44–47]. The local indicators of spatial analysis (LISA) cluster maps also illustrate that there are hot-spot areas on the border of Jarar and Fafan zones of Somalia with Somali-land, Liben zone of Somalia region, Borena zone of Oromiya region with Kenya, Metekel zone of Benishangul-Gumuz, and West Gondar zone of Amhara region with Sudan at a different time in the study period. These findings are consistent with the findings of the current literature [14, 18, 44], suggesting that there is a connection between TB transmission and territorial or international borders. Therefore, in order to achieve a global solution and targeted intervention, it is important to expand the nation-level study to higher spatial dimensions that encompass at least neighboring countries [50–52].

Whereas in 2015 hot-spots for the prevalence of HIV among tuberculosis patients were observed in some districts of Afar, Amhara, Gambela, Oromiya, Somali regions, and Addis Ababa city administration. In addition, to those regions which had hot-spot districts in 2015, in 2016, hot-spots were also observed in districts of the Tigray region. However, in 2017 except in Gambella region and Addis Ababa city administration those regions which had hot-spots for the prevalence of TB among PLHIV also had hot-spot districts for the prevalence of HIV among TB patients. Hot-spots were also observed for HIV among TB patients in districts of Afar, Amhara, Oromiya, Somali, Tigray regions and districts of Addis Ababa city administration in 2018. However, more districts in the Oromiya region in 2015, Benishangul-Gumuz and Oromiya regions in 2016, Benishangul-Gumuz, Gambela, Oromiya, and SNNP regions in 2017, and Benishangul-Gumuz, Harer, Oromiya and SNNP regions, and in Dire Dawa city administration in 2018 were identified as cold-spots, respectively.

Hot-spot areas for the prevalence of HIV among TB patients were also observed at the border of Nuer and Anuak zones in Gambella region with South Sudan, Jarar zone of Somalia region with Somali-land, Shabelle and Afder zones of Somalia region with Somalia, Eastern Tigray with Eritrea, and Awsi Rasu zone of Afar with Djibouti. There are refugee camps close to the border of Gambella and Tigray regions and in some areas which are the border of Oromiya and Somalia regions and Kenya. Generally, refugees are often in situations where they do not have a proper job access to generate additional income. As a result, women and young girls in refugee camps often enter commercial sex work to earn income, food, and to gain access to other resources [53]; this exposes them to HIV/AIDS and other sexually transmitted diseases (STDs) infections. Furthermore, in these areas HIV vulnerability can raise as districts in these areas have poor access to health-care facilities and integrated service provision to address TB-HIV coinfection [18].

According to the findings of the current study, over the majority of the study period in Ethiopia, the prevalence of HIV among TB patients was more geographically associated than the prevalence of TB among PLHIV. This finding agrees with Aturinde et al. [54] where they found that HIV was more geographically associated than TB in Uganda for the period 2015–2017. The global Moran's *I* statistics for TB among PLHIV, except in 2018, and HIV among TB patients were positive suggesting that neighboring districts tend to possess similar characteristics in the prevalence of TB/HIV coinfection. The global bivariate Moran's *I* statistic was also positive for the study period, implying that neighboring districts had an effect on the two types of TB-HIV coinfection. Spearman's correlation coefficients were positive and statistically significant, they are in agreement with Moran's *I* statistic values. These coefficients are more than 0.5 for each year and they are consistent with results reported in [54, 55].

Despite the fact that there are a limited number of studies that have examined the spatial clustering of HIV and TB separately at the national level in Ethiopia [56] and TB-HIV coinfection [18]; to the best of the authors' knowledge, this is the first spatial analysis that considered TB-HIV coinfection using yearly data. However, there were some limitations that might have had an impact on our results. First, because the data were aggregated at the district level, the results of this study cannot be generalized to small administrative units of the country or Kebele, household, or individual level. Second, due to under-reporting or underdetection of cases, the number of notified HIV and TB cases may not accurately reflect the burden of the diseases in a district because; in this study, data on HIV cases among TB patients and TB among PLHIV were gathered from the national HMIS electronic surveillance system. For example, symptomatic people who did not receive HIV or TB diagnosis and treatment may not be reported. If spatial generalized linear models, specifically employing count models to evaluate potential risk factors for TB-HIV coinfection, are used to corroborate the findings, the findings could be more intriguing.

## 5. Conclusion

In this study, we evaluated the spatial clustering of two types of TB-HIV coinfection prevalence by applying the global Moran's *I* statistic, Getis–Ord *G*_*i*_^*∗*^ statistic with LISA cluster maps and Spearman correlation coefficient with its modified *t*-test. According to the findings of our study, between the years of 2015 and 2018, there was strong spatial clustering in Ethiopia of both the prevalence of HIV among TB patients and the prevalence of TB among PLHIV at the districts level. The LISA detected districts which were hot-spots and cold-spots for the prevalence of TB among PLHIV and the prevalence of HIV among TB patients in various regions and the two city administrations. In some cases, these spots were unstable over the study period except for some of the districts in the Afar and Amhara regions and Addis Ababa city administration which were consistently identified as hot-spots for the prevalence of HIV among TB patients. The study presents detailed knowledge about the spatial clustering of TB-HIV coinfection in Ethiopia at the districts level and the findings could provide information for planning coordinated district-specific interventions to jointly control both diseases in Ethiopia.

## Figures and Tables

**Table 1:** The number of TB, HIV, and TB/HIV coinfected patients reported in Ethiopia by gender and year.

Variable^1^	Male	Female	Total
2015			
Number of TB cases enrolled to DOTS and who were tested for HIV	49,949	41,433	91,382
Number of TB patients with HIV-positive test result	3,804	3,382	7,186
Number of patients enrolled in HIV care who were screened for TB during their visit	239,139	384,788	623,927
Total number of HIV patients with active TB	1,880	1,723	3,603

2016			
Number of TB cases enrolled to DOTS and who were tested for HIV	49,802	41,890	91,692
Number of TB patients with HIV-positive test result	3,528	3,233	6,761
Number of patients enrolled in HIV care who were screened for TB during their visit	348,148	566,839	914,987
Total number of HIV patients with active TB	2,097	1,775	3,872

2017			
Number of TB cases enrolled to DOTS and who were tested for HIV	51,564	41,753	93,317
Number of TB patients with HIV-positive test result	3,391	2,891	6,282
Number of patients enrolled in HIV care who were screened for TB during their visit	389,650	651,680	1,041,330
Total number of HIV patients with active TB	1,899	1,788	3,687

2018			
Number of TB cases enrolled to DOTS and who were tested for HIV	28,991	23,411	52,402
Number of TB patients with HIV-positive test result	1,988	1,616	3,604
Number of patients enrolled in HIV care who were screened for TB during their visit	266,948	452,703	719,651
Total number of HIV patients with active TB	1,228	1,503	2,731

^1^HIV: human Immunodeficiency virus; TB: tuberculosis; DOTS: directly observed therapy, short course.

**Table 2:** The prevalence of HIV among TB patients (HIV(TB)) and the prevalence of TB among HIV patients (TB(HIV)) by year in all regions and of Ethiopia.

	Year							
	2015		2016		2017		2018	
Region/city^2^	HIV (TB)	TB (HIV)	HIV (TB)	TB (HIV)	HIV (TB)	TB (HIV)	HIV (TB)	TB (HIV)
Addis Ababa	24.89	0.83	24.98	0.55	22.40	0.55	20.76	0.44
Afar	7.58	4.00	7.54	1.89	5.12	1.54	5.08	1.42
Amhara	8.76	0.34	7.82	0.29	6.99	0.20	7.02	0.34
Beneshangul Gumuz	7.37	0.74	5.32	0.54	3.23	1.42	3.40	0.44
Dire Dawa	12.75	0.50	15.22	0.55	10.55	0.56	11.72	0.39
Gambela	31.59	5.17	18.01	13.71	13.32	3.73	9.72	1.44
Harari	6.25	38.32	9.27	0.22	13.02	0.31	13.06	0.25
Oromiya	5.56	0.50	4.62	0.34	4.44	0.31	3.98	0.31
SNNP	4.05	3.57	4.19	1.51	3.09	0.76	3.58	0.56
Somali	6.78	100.00	7.40	21.60	19.86	28.57	15.49	0.00
Tigray	9.12	0.91	8.71	0.23	8.31	0.36	8.00	0.63
Ethiopia	7.86	0.56	7.37	0.42	6.73	0.35	6.88	0.38

^2^Two city administrations: Addis Ababa and Dire Dawa.

**Table 3:** Moran's I statistic (*p* value) for HIV among TB patients (HIV) and TB among people living with HIV (TB) by year.

	Year			
	2015	2016	2017	2018
*Global Moran's I*				
TB (HIV)	0.069 (<0.001)	0.009 (0.710)	0.037 (<0.001)	−0.001 (0.9300)
HIV (TB)	0.118 (<0.001)	0.134 (<0.001)	0.021 (<0.001)	0.067 (<0.001)

*Bivariate global Moran's I*				
TB/HIV	0.152 (<0.001)	0.251 (<0.001)	0.230 (<0.001)	0.224 (<0.001)

*Spearman's correlation*				
TB/HIV	0.558 (<0.001)	0.692 (<0.001)	0.633 (<0.001)	0.517 (<0.001)

## Data Availability

The data that support the findings of this study are available from Ethiopian Ministry of Health Office but restrictions apply to the availability of these data, which were used under license for the current study, and so are not publicly available. Data are, however, available from the authors upon reasonable request and with permission of Ethiopian Ministry of Health Office.

## Abbreviations

AIDS: Acquired immunodeficiency syndrome
DOTS: Directly observed therapy, short course
HMIS: Health management information system
HIV: Human immunodeficiency virus
LISA: Local indicators of spatial analysis
STDs: Sexually transmitted diseases.
